# Activation of mitogen-activated protein kinases in satellite glial cells of the trigeminal ganglion contributes to substance P-mediated inflammatory pain

**DOI:** 10.1038/s41368-019-0055-0

**Published:** 2019-09-10

**Authors:** Yanyan Zhang, Ning Song, Fei Liu, Jiu Lin, Mengke Liu, Chaolan Huang, Daqing Liao, Cheng Zhou, Hang Wang, Jiefei Shen

**Affiliations:** 1 State Key Laboratory of Oral Diseases, National Clinical Research Center for Oral Diseases, West China Hospital of Stomatology, Sichuan University, Chengdu, China; 2 West China School of Stomatology Sichuan University, Chengdu, China; 3 Laboratory of Anesthesia & Critical Care Medicine, Translational Neuroscience Center, West China Hospital of Sichuan University, Chengdu, China

**Keywords:** Extracellular signalling molecules, Molecular medicine

## Abstract

Inflammatory orofacial pain, in which substance P (SP) plays an important role, is closely related to the cross-talk between trigeminal ganglion (TG) neurons and satellite glial cells (SGCs). SGC activation is emerging as the key mechanism underlying inflammatory pain through different signalling mechanisms, including glial fibrillary acidic protein (GFAP) activation, phosphorylation of mitogen-activated protein kinase (MAPK) signalling pathways, and cytokine upregulation. However, in the TG, the mechanism underlying SP-mediated orofacial pain generated by SGCs is largely unknown. In this study, we investigated whether SP is involved in inflammatory orofacial pain by upregulating interleukin (IL)-1β and tumour necrosis factor (TNF)-α from SGCs, and we explored whether MAPK signalling pathways mediate the pain process. In the present study, complete Freund’s adjuvant (CFA) was injected into the whisker pad of rats to induce an inflammatory model in vivo. SP was administered to SGC cultures in vitro to confirm the effect of SP. Facial expression analysis showed that pre-injection of L703,606 (an NK-1 receptor antagonist), U0126 (an inhibitor of MAPK/extracellular signal-regulated kinase [ERK] kinase [MEK] 1/2), and SB203580 (an inhibitor of P38) into the TG to induce targeted prevention of the activation of the NK-1 receptor and the phosphorylation of MAPKs significantly suppressed CFA-induced inflammatory allodynia. In addition, SP promoted SGC activation, which was proven by increased GFAP, p-MAPKs, IL-1β and TNF-α in SGCs under inflammatory conditions. Moreover, the increase in IL-1β and TNF-α was suppressed by L703, 606, U0126 and SB203580 in vivo and in vitro. These present findings suggested that SP, released from TG neurons, activated SGCs through the ERK1/2 and P38 pathways and promoted the production of IL-1β and TNF-α from SGCs, contributing to inflammatory orofacial pain associated with peripheral sensitization.

## Introduction

Orofacial pain, referring to a cluster of disorders including trigeminal neuralgia, temporomandibular joint disorders, migraine and orthodontic pain, affects 16% of the general population and causes a dramatic reduction in the quality of life.^1,2^ Orofacial inflammation can alter the properties of somatic sensory pathways, resulting in pain abnormalities such as hypersensitivity, hyperalgesia and allodynia.^3^ As the key relay station in the peripheral pathway of orofacial pain, the trigeminal ganglion (TG) contains the somas of sensory TG neurons and surrounding satellite glial cells (SGCs).^4–6^ Current evidence has revealed that SGCs play an important role in the peripheral sensitization of the TG.^7^ The profound cross-talk network between TG neurons and SGCs is essential to the regulation of inflammatory orofacial pain.^8,9^

Several chemical messengers take part in this process, including substance P (SP), adenosine triphosphate (ATP), calcitonin gene-related peptide (CGRP), and γ-aminobutyric acid (GABA).^10,11^ For instance, TG neurons synthesize and secrete more SP following peripheral inflammation, which activates the neurokinin (NK)-1 receptors on SGCs to trigger local paracrine mechanisms.^8,12^ In the dorsal root ganglion (DRG) of the spinal cord, there is evidence that activated SGCs and the subsequent production of cytokines, such as interleukin (IL)-1β^13^ and tumour necrosis factor (TNF)-α,^14,15^ contribute to the development and maintenance of chronic neuropathic pain. It has also been shown that IL-1β is increased in the TG under conditions of inflammation and nerve injury.^16^ However, in SGCs of the TG, the mechanism underlying SP-mediated processes has been largely unclear.

Activation of mitogen-activated protein kinases (MAPKs), including extracellular signal-regulated kinase (ERK), P38, and c-Jun N-terminal kinase (JNK),^17^ in DRG neurons of the spinal cord by peripheral noxious stimulation leads to pain hypersensitivity.^8,17^ It has also been reported that the activation of ERK in SGCs of the TG is involved in lingual neuropathic pain.^18^ However, the roles of ERK, P38, JNK and their downstream regulators in SGCs during the regulation of inflammatory orofacial pain remain unclear.

While previous studies on the effects of SP and the intracellular signalling pathways have mainly focused on the sensory neurons in the DRG and TG, in this study, we aimed to investigate the effect of SP on the intracellular MAPK pathway and cytokine production in SGCs during the peripheral activation process of orofacial pain. Our results demonstrated that SP induced the production of IL-1β and TNF-α in TG SGCs by activating NK-1 receptors and MAPKs involved in these processes.

## Results

### Inflammation-induced allodynia and inhibitors of the MAPK signalling pathway attenuated allodynia

**Fig. 1:**
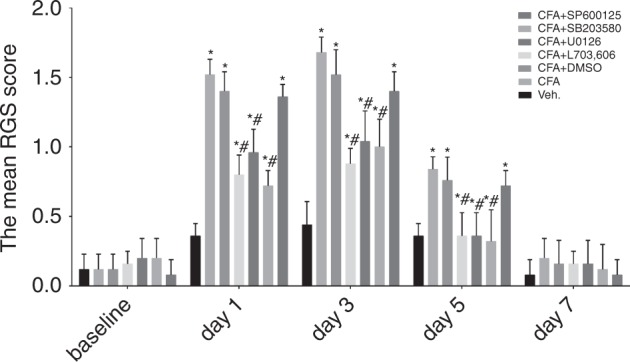
Changes in RGS score following the administration of the vehicle and different drugs (vehicle, CFA, CFA+DMSO, CFA+L703, 606, CFA+U0126, CFA+SB203580 and CFA+SP600125) in rats in vivo. The RGS scores of the CFA group increased on day 1, peaked on day 3, started to decrease on day 5, and returned to baseline on day 7, and these scores were all higher than those in the vehicle group. The data showed that in the CFA+L703, 606, CFA+U0126 and CFA+SB203580 groups, the RGS scores increased on day 1, peaked on day 3 but were still significantly lower than those in the CFA group, and returned to baseline on days 5–7. (*n* = 5, *P < 0.05 compared with the vehicle group, ^#^*P* < 0.05 compared with the CFA group)

In this study, the changes in the allodynia levels of rats were assessed using the RGS score. Images captured from videotapes are listed in Supplementary Fig. 1. According to the RGS scores, we demonstrated the following findings. The RGS scores of the CFA group increased on day 1, peaked on day 3, started to decrease on day 5 but was still higher than the scores of the vehicle group, and returned to baseline on day 7 (Fig. 1, *n* = 5, *P* < 0.05). There was no significant difference among the CFA+DMSO, CFA+SP600125 and CFA groups during the entire observation period (Fig. 1, *n* = 5, *P* > 0.05), indicating that DMSO is a safe solvent with no side effects and that SP600125 has no therapeutic effect on allodynia in rats. However, in the CFA+L703, 606, CFA+U0126 and CFA+SB203580 groups, the RGS scores increased on day 1, peaked on day 3 but were still significantly lower than the scores in the CFA group, and returned to baseline on days 5–7 (Fig. 1, *n* = 5, *P* < 0.05), meaning that U0126 and SB203580 can attenuate the inflammatory pain mediated by SP.

### Expression of NK-1 receptors on SGCs in the TG

**Fig. 2:**
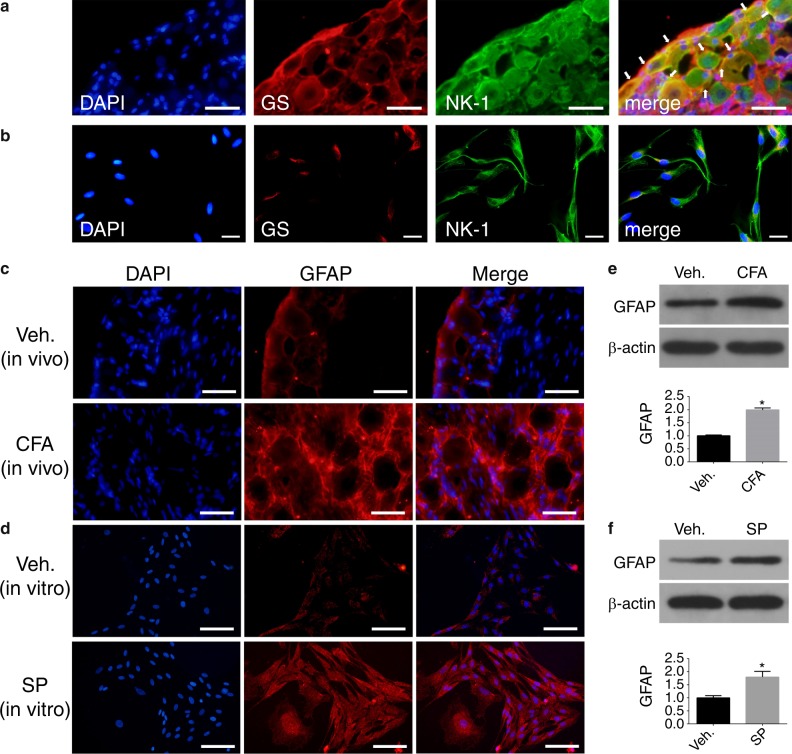
IF staining of the NK-1 receptor and the changes in GFAP in TGs and SGC cultures. **a**, **b** IF staining of the NK-1 receptor in rat TG slices and SGC cultures. (green: NK-1; red: GS; blue: DAPI). **c**, **d** IF staining of GFAP on rat TG slices and SGC cultures. (red: GFAP; blue: DAPI). Scale bars: 50 μm. The representative WB images and the bar graph shown in (**e**) illustrated that the expression of GFAP in the CFA-treated groups was higher than that in the vehicle-treated groups. The representative WB images and the bar graph shown in (**f**) illustrate that the expression of GFAP in SP-treated SGCs was higher than that in vehicle-treated SGCs. (*n* = 6, *P < 0.05 compared with the vehicle group)

To confirm that SP mediates the activation of SGCs via the NK-1 receptor, the expression of the NK-1 receptor was detected in TG and SGC cultures. In our study, GS was used as a specific marker for SGCs to analyse the location of NK-1 receptors. As shown in Fig. 2a, TG neurons were encircled with GS-IR cells. Both SGCs and neurons expressed NK-1 receptors. NK-1 receptor and GS staining were co-localized on SGCs (arrows). The IF results of the SGC cultures in vitro also supported NK-1 receptor expression on SGCs (Fig. 2b). These findings indicated that SP regulated the function of SGCs by activating the NK-1 receptor on SGCs, which was consistent with the findings of a previous study.^12^

### SP-induced upregulation of GFAP in SGCs under inflammatory conditions

GFAP expression is related to the activation of glial cells.^19^ To investigate whether SGCs are activated by SP under inflammatory conditions, the expression of GFAP, a special marker of SGC activation,^16,20^ was analysed by IF staining and WB in vivo and in vitro. The IF analysis results showed that GFAP-IR SGCs were observed in TG slices and SGC cultures (Fig. 2c, d). The percentage of GFAP-IR SGCs in the TG of the CFA group was significantly higher than that in the vehicle group in vivo (Fig. 2c, *n* = 3, *P* < 0.05). The WB results confirmed that 24 h after CFA injection, GFAP expression in the CFA-treated group was significantly increased compared with that in the vehicle group (Fig. 2e, *n* = 6, *P* < 0.05). Then, we measured GFAP changes in SGC cultures in vitro to further verify the activation of SGCs (Fig. 2d, f, *n* = 6, *P* < 0.05). A similar pattern was also observed in SP-treated SGC cultures. Therefore, the findings in our study were consistent with those in previous studies showing that GFAP was upregulated in SGCs under inflammatory conditions,^19,21,22^ further inferring that the activation of SGCs might play an important role in inflammatory orofacial pain.

### SP upregulated the phosphorylated MAPKs in SGCs under inflammatory conditions

**Fig. 3:**
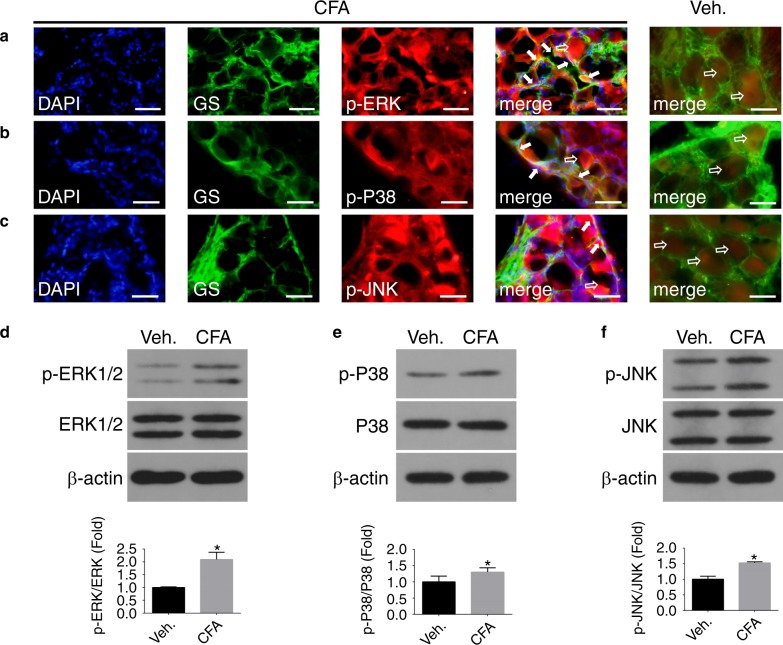
The expression of p-MAPKs in vivo after vehicle administration and 24 h after CFA injection. **a**–**c** IF staining of p-MAPKs in rat TG slices in the vehicle and CFA groups. Arrows (solid) show that p-MAPKs co-localized with GS, and arrows (hollow) show that p-MAPKs localized in TG neurons (green: GS; red: p-ERK1/2, p-P38, and p-JNK; blue: DAPI). Scale bars: 50 μm. WB images and graphs in **d**–**f** show that the expression of p-ERK1/2, p-P38 and p-JNK in CFA-treated TGs is higher than that in vehicle TGs, and these levels were normalized against their total levels. (*n* = 6, *P < 0.05 compared with the vehicle group)

**Fig. 4:**
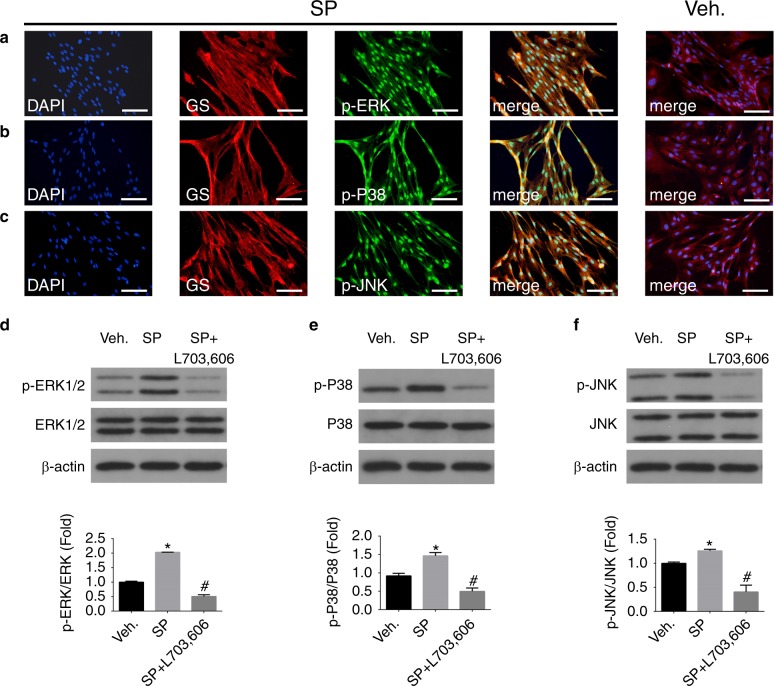
The expression of p-MAPKs on SGCs in vitro after vehicle administration and 24 h after SP and L703, 606 administration. **a–c** IF staining of p-MAPKs in the vehicle and SP groups. (green: p-ERK1/2, p-P38, and p-JNK; red: GS; blue: DAPI). Scale bars: 100 μm. WB images and graphs in **d–f** show the quantification of p-ERK1/2, p-P38 and p-JNK expression levels in vehicle-, SP- and SP+L703, 606-treated SGCs for 24 h. SP pretreatment upregulated the expression of p-MAPKs in SGCs, and L703,606 abolished the effect of SP. (*n* = 6, *P < 0.05 compared with the vehicle group, ^#^*P* < 0.05 compared with SP group)

Activation of NK-1 receptors on SGCs can trigger various cellular changes.^12^ To investigate the effect of SP on MAPK signalling pathways in SGCs, TGs from CFA-treated animals and SGC cultures administered with SP were obtained to examine the phenotypical expression of p-ERK1/2, ERK1/2, p-P38, P38, p-JNK and JNK. As shown in Fig. 3a, p-ERK staining was readily detectable both in TG neurons (hollow arrows) and in SGCs (solid arrows) in CFA-treated TGs. Similar to the results of p-ERK, the expression levels of p-P38 and p-JNK were readily observed in TG neurons and in SGCs in CFA-treated TGs (Fig. 3b, c). Almost no p-MAPKs were expressed on SGCs in the vehicle group, but the expression levels of p-MAPKs were significantly increased in the CFA group. In CFA-injected rats, the immunoreactivity for p-ERK, p-P38 and p-JNK was also increased in TG neurons. Furthermore, the levels of ERK, p-ERK, P38, p-P38, JNK, and p-JNK in the vehicle and CFA groups were analysed by WB. The levels of p-ERK1/2, p-P38 and p-JNK were normalized against their total expression levels. According to the WB results, low levels of p-ERK, p-P38 and p-JNK were observed in the vehicle group; however, an approximately 2-fold increase was detected in response to CFA treatment in vivo (Fig. 3d–f, *n* = 6, *P* < 0.05), which indicated that CFA induced the activation of ERK1/2, P38 and JNK MAPK signalling in the TG. To verify that the abovementioned phosphorylation of the MAPK pathway in SGCs was induced by phosphorylated SP, which was highly secreted by TG neurons in a CFA-induced inflammation model, total MAPKs were examined in SGC cultures in vitro. The initial screening of MAPK involvement in SP-related activation of SGCs was performed using IF staining and WB. IF staining showed phenotypically high expression of p-ERK, p-P38 and p-JNK MAPKs both in the cytosol and nuclei of SGCs in response to SP-induced inflammation compared to the vehicle SGCs (Fig. 4a–c). For WB analysis, similar results were observed in SP-treated SGC cultures. The results also indicated that L703, 606 (an antagonist of the NK-1 receptor) inhibited the upregulation of p-ERK, p-P38 and p-JNK (Fig. 4d–f, *n* = 6, *P* < 0.05).

### SP upregulated the production of IL-1β and TNF-α in SGCs under inflammatory conditions

**Fig. 5:**
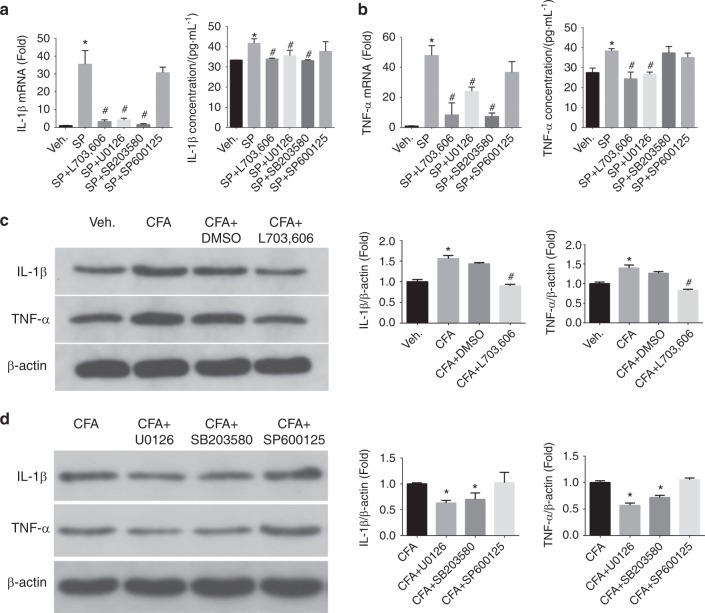
Changes in IL-1β and TNF-α mRNA and protein levels in SGCs after different administrations in vitro (vehicle, SP, SP+L703, 606, SP+U0126, SP+SB203580 and SP+SP600125) for 24 h (**a**, **b**) and in TGs after different treatments in vivo (vehicle, CFA, CFA+DMSO, CFA+L703, 606, CFA+U0126, CFA+SB203580 and CFA+SP600125 at 24 h (**c**, **d**). Up/downregulation compared with vehicle and SP treatments in vitro and with the vehicle and CFA groups in vivo. According to the RT-qPCR and ELISA results (**a**), there was a strong and significant upregulation of IL-1β mRNA in SGCs compared to the vehicle treatment. Conversely, suppression of the SP-induced increase in IL-1β by L703.606, U0126, and SB203580 was significant. (*n* = 6, *P < 0.05 compared with the vehicle group. ^#^*P* < 0.05 compared with SP group). (**b**) In addition, there was a strong and significant upregulation of TNF-α mRNA in SGCs compared to the vehicle. Suppression of the SP-induced increase in TNF-α by L703.606, U0126 and SB203580 was significant. The ELISA results showed that the inhibitory effect of SB203580 was not significant. (*n* = 6, *P < 0.05 compared with the vehicle group, ^#^*P* < 0.05 compared with SP group). **c** Representative WB images and graphs illustrate that the production of IL-1β and TNF-α in rat TGs was significantly increased under CFA injection compared to the vehicle. Pre-injection of L703, 606 into TGs significantly reduced the upregulation of IL-1β and TNF-α induced by CFA. (*n* = 6, *P < 0.05 compared with the vehicle group, ^#^*P* < 0.05 compared with CFA group). Pre-injection of U0126 and SB203580 into TGs also significantly reduced the upregulation of IL-1β and TNF-α induced by CFA. (*n* = 6, *P < 0.05 compared with the CFA group)

To investigate the effect of SP on the production of IL-1β and TNF-α in SGCs, we designed experiments in vitro and in vivo. In vitro, we incubated the SGCs in vehicle (0.5% DMSO), SP (1 μmol·L^–1^) and SP (1 μmol·L^–1^) +L703, 606 (1 μmol·L^–1^) for 24 h. According to the RT-qPCR results, the IL-1β mRNA was increased approximately 35-fold after SP stimulation compared to after administration of the vehicle. SP-induced upregulation of IL-1β mRNA was blocked by L703, 606 (an NK-1 receptor antagonist) (Fig. 5a, *n* = 6, *P* < 0.05). The ELISA results showed similar trends (Fig. 5a, *n* = 6, *P* < 0.05). Likewise, TNF-α mRNA was significantly higher after SP stimulation, with an approximately 45-fold increase compared with after administration of the vehicle. L703, 606 reversed the SP-induced increase in TNF-α mRNA (Fig. 5b, *n* = 6, *P* < 0.05). Similar results from the ELISA assays showed increased TNF-α expression as mentioned above (Fig. 5b, *n* = 6, *P* < 0.05). We further designed experiments in vivo, including with the use of vehicle, CFA, CFA+DMSO and CFA+L703, 606 groups, to further verify that SP is involved in the mediation of IL-1β and TNF-α production in TGs under inflammatory conditions. There were similar findings in vivo according to WB analyses. IL-1β and TNF-α were significantly upregulated under inflammatory conditions in rat TGs, and administration of the vehicle, and L703, 606 inhibited this upregulation (Fig. 5c, *P* < 0.05, *n* = 6). These findings indicated that SP mediated the changes in IL-1β and TNF-α in TGs under CFA-induced inflammatory conditions.

### The effects of MAPK signalling pathways on the SP-induced production of IL-1β and TNF-α in SGCs

The roles of MAPKs in the upregulation of mRNA and protein levels of IL-1β and TNF-α from SGCs were assessed by testing the effects of inhibitors of MAPKs in vitro and in vivo. SGCs were incubated in SP alone or were co-cultured with inhibitors to establish the following groups: vehicle, SP, SP+U0126, SP+SB203580 and SP+SP600125. Suppression of the SP-induced increase in IL-1β by U0126 and SB203580 was significant. However, at the dose of SP600125 we used, there was no obvious inhibitory effect (Fig. 5a, *n* = 6, *P* < 0.05). In addition, according to the RT-qPCR results, suppression of the SP-induced increase in TNF-α by U0126 and SB203580 was significant. Nevertheless, there was no obvious inhibitory effect in the SP600125 groups (Fig. 5b, *n* = 6, *P* < 0.05). However, based on the ELISA results, the inhibitory effect of SB203580 was not significant. To verify that the MAPK pathway is involved in the upregulation of IL-1β and TNF-α under inflammatory conditions induced by CFA, inhibitors of MEK, P38 and JNK were administered into TGs. The WB results were similar to the findings in vitro (Fig. 5d, *n* = 6, *P* < 0.05).

## Discussion

Orofacial pain is highly associated with the peripheral sensitization of the TG.^2^ Recently, it has been increasingly appreciated that not only neurons but also glial cells play essential roles in enhancing and maintaining inflammatory pain.^23^ TG neurons synthesize and secrete more SP following inflammation,^12^ and SP plays an important role in promoting peripheral activation.^24^ MAPKs also participate in the activation of multiple glial receptors and the production of pro-inflammatory and pro-nociceptive mediators,^25^ which mediate the process of inflammatory pain. However, whether MAPKs participate in SP-mediated SGC activation is largely unknown. In this study, we found that inflammatory orofacial allodynia in rats was related to SP. SP was involved in the activation of SGCs, promoted the production of pro-inflammatory cytokines (IL-1β and TNF-α) in SGCs via the NK-1 receptor and upregulated the phosphorylation of the MAPK signalling pathway in vivo and in vitro under inflammatory conditions. It was also found that the application of inhibitors of ERK and P38 pathways attenuated the orofacial allodynia induced by inflammation and inhibited the increase in IL-1β and TNF-α triggered by SP. In summary, SP led to the activation of SGCs, which play important roles in the regulation of inflammatory orofacial pain.

SP has a variety of physiological effects in regulating the immune system, affecting the reproductive and endocrine systems.^26^ SP participates in the production and maintenance of pain in the nervous system by acting as an important neurotransmitter.^26^ Both in vitro and in vivo studies have revealed that SP is synthesized and secreted from the neuronal soma, which secretes a series of molecules activating neurons and SGCs, and SP activates NK-1 receptors in SGCs under inflammatory conditions, thus stimulating the production of interleukin IL-1β in glial cells from TGs.^27^ NK-1 antagonists have been shown to reduce inflammatory pain in several different animal models,^28^ including the NK-1 knockout mouse model.^29^ We also obtained evidence that blockade of the NK-1 receptor within the TG can attenuate the sensitization of TG neurons.^27^ Given the pivotal role of SP in exerting neuralgia, our experiments aimed at investigating the effect of SP on TG SGCs. We found that both neurons and SGCs in TGs expressed the NK-1 receptor in vivo and in vitro, which was similar to the findings of previous studies.^12^ The increased level of NK-1 receptors on SGCs after inflammation indicated that this receptor was activated by SP stimuli.^30^ SP participated in the regulation of orofacial allodynia under inflammatory conditions induced by CFA, which was in agreement with the findings of the previous work.^31^ It was also found that allodynia was attenuated by pre-injection of L703, 606 (an antagonist of the NK-1 receptor) into the TG, suggesting that SP mediates inflammatory allodynia by activating the NK-1 receptor.

SGCs have strong structural interrelationships with neurons, and this is the basis of cross-talk between glia and neurons. Neurons and SGCs form a functional unit within the TG and are involved in modulating the excitability of neurons by “listening” and “talking” to neurons.^28^ SGC activation involves the modulation of TG neuron excitability and plays an essential role in enhancing and maintaining facial pain.^23,28^ Evidence suggests that glial cells are activated by ionic changes, post-translational regulation, morphological changes and proliferation.^3^ We focused on TG SGCs and found that SP led to the activation of SGCs under inflammatory conditions in vivo and in vitro, as evidenced by an increase in GFAP expression in SGCs (a specific marker of activated SGCs).^32^ Our findings were consistent with the fact that higher levels of GFAP were detected in the SGCs under different conditions, for example, nerve injury, inflammation, joint arthritis, bone cancer and chemotherapy.^3^

The secretion of pro-inflammatory cytokines (IL-1β and TNF-α) is increased by many cell types, including astrocytes and microglia, under stress, injury, or inflammatory conditions.^33^ IL-1β heightens the neuronal excitability of TG neurons by suppressing the voltage-gated potassium currents in TG neurons via protein kinase C/G protein-coupled signalling pathways by regulating IL-1RI,^20,22,28^ thereby promoting the formation and maintenance of oral and maxillofacial hyperalgesia and/or allodynia. Previous studies have shown that TNF-α plays a role in orofacial pain by regulating TRPV1, TNFR1, TNFR2 and Cdk5, which are expressed in TG neurons.^34,35^ Given the important effects of IL-1β and TNF-α, special consideration has been given to these factors in the current research of SP-induced SGC activation. In our study, clear responses of TG SGCs to SP were observed. The RT-qPCR, ELISA and WB measurements were used to detect the levels of IL-1β and TNF-α in vivo and in vitro. We found that SP could promote the production of IL-1β and TNF-α in SGCs under inflammatory conditions, which was in partial agreement with previous results obtained in the TG.^16^ The pre-induction of an NK-1 receptor antagonist (L703, 606) into the TG in vivo and into SGC cultures prevented the upregulation of IL-1β and TNF-α. Taken together, these data suggested that SP caused an increase in IL-1β and TNF-α by activating the NK-1 receptor.

Accumulating evidence has implicated that elevated levels of p-ERK in DRG and TG neurons,^36^ as well as SGCs,^18^ contribute to the development of peripheral sensitization.^37,38^ The MAPK signalling pathway participates in cellular functions such as proliferation, differentiation, migration and apoptosis, as well as chronic inflammation.^39^ In particular, the expression of p-P38 and p-ERK in SGCs and of p-JNK^40^ in astrocytes in the spinal cord leads to the induction of pro-inflammatory genes, including IL-1β, IL-6, prostaglandin (PG)E2, and TNF-α, thus contributing to central sensitization.^41^ Considering the essential functions of the MAPKs mentioned above, we propose that MAPKs might contribute to the peripheral sensitization of TG SGCs. We observed the following findings. SP significantly enhanced p-ERK, p-P38 and p-JNK in rat TG SGCs under inflammatory conditions in vivo and in vitro. Our results were not exactly the same as some previously published studies. Some studies reported that the activation of ERK and P38 but not JNK signalling expression was reinforced following paclitaxel treatment^42^ in the DRG and TG. On the other hand, the activation of JNK in astrocytes involved in mechanical allodynia under inflammatory conditions was also proposed.^43^ Evidence has also suggested that only p-ERK is upregulated in nerve injury, whereas p-P38 and p-JNK are not.^3^

The contribution of MAPKs to the SP-induced changes in behavioural pain levels was assessed by testing the effects of MAPK inhibitors on the facial expression of rats in vivo. In the present study, pretreatment with U0126 (an inhibitor of MEK1/2) and SB203580 (an inhibitor of P38) into the TG attenuated CFA-induced inflammatory allodynia. The contribution of MAPKs to the SP-induced changes in IL-1β and TNF-α levels in SGCs was assessed by testing the effects of MAPK inhibitors in vivo and in vitro. According to the results, U0126 and SB203580 suppressed IL-1β and TNF-α at the mRNA and protein levels. The findings were consistent with the former conclusion, which reported that inhibitors of MEK1/2 and P38 but not JNK prevented paclitaxel-enhanced hypersensitivity.^42^ To summarize the findings above, not all antagonists expressed an inhibitory effect. The reason for this inconsistency is not clear. Previous reports have suggested that glial cells might produce both inflammatory signalling molecules and anti-inflammatory and anti-nociceptive mediators.^3^ These mediators may play a crucial role in the inflammatory pain system and may regulate downstream effects, such as those shown in our experimental results. Furthermore, the three major MAPK pathways may have mutual effects on the modulation of pain mechanisms. There are certain degrees of self-regulatory mechanisms in the nervous system. When one of the pathways is selectively inhibited, the other pathways may be retrospectively enhanced, thereby affecting the levels of downstream signals. Taken together, these findings support the essential roles of ERK and P38 in regulating the effect of SP on the production of pro-inflammatory cytokines in SGCs and the induction and maintenance of inflammatory pain.

**Fig. 6:**
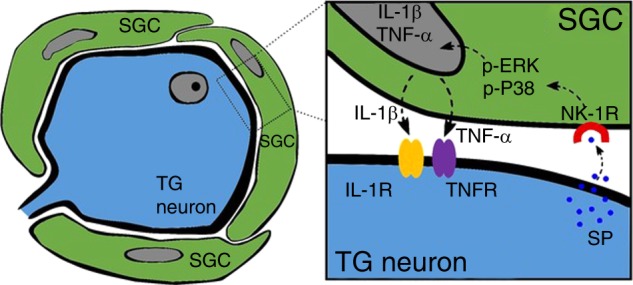
Schematic representation of a possible molecular mechanism involved in the effect of SP on the cytokine production of pro-inflammatory cytokines in TG SGCs. SP, secreted by TG neurons, causes SGC activation by activating NK-1 receptors expressed on the surface of SGCs, thus causing important downstream effects including upregulating the secretion of IL-1β and TNF-α by activating ERK and P38 MAPK pathways in SGCs. Then, IL-1β and TNF-α activate their specific receptors (IL-1R and TNFR) in TG neurons, resulting in enhanced cross-talk between TG neurons and SGCs, which plays an important role in inflammatory orofacial pain

The TG is a complex modulation system consisting of neurons, SGCs, Schwann cells, immune cells, and macrophages.^17,18,44^ These cells may have been involved in the regulation of orofacial pain in vivo in this experiment. Many chemical messengers, such as SP, CGRP, BDNF, and ATP, are released in the TG and may activate SGCs and neurons through MAPK signalling pathways as well as contribute to the release of IL-1β and TNF-α under inflammatory conditions induced by CFA. MAPK activation not only in SGCs but also in sensory neurons may contribute to behavioural hypersensitivity in vivo.^18^ NK1 receptors are expressed in both neurons and SGCs, so SP may also activate neurons in vivo. Therefore, we cannot rule out the roles of the abovementioned messengers and neurons in orofacial pain in vivo. We designed a study of SGC cultures in vitro using the simple treatment of SP to mainly study the effect of SP on SGC activation. L703,606 was used to inhibit the function of the NK-1 receptor to further verify the role of SP. Although we determined that the purity of the SGC culture used was above 95% according to GS staining, the presence of macrophages could not be ruled out since IL-1β, TNF-α and other chemical mediators can also be released from macrophages and other immune cells under inflammatory conditions.^45^ Due to the structural characteristics of neurons and SGCs, SGCs alone cannot induce pain; instead, they serve as pain amplifiers to enhance pain. IL-1β and TNF-α enhanced the excitability of neurons by activating their specific receptors (IL-1R and TNFR), which are expressed on TG neurons, thus promoting inflammatory pain (Fig. 6).^20,22,34,35^

In conclusion, SGC activation, which was proven by increased GFAP expression and the phosphorylation of MAPK expression induced by SP under inflammatory conditions, is involved in the maintenance of orofacial pain. Other cytokines, chemokines, and inflammatory mediators in neurons, SGCs, immune cells and macrophages also need to be taken into consideration for participation in the production and maintenance of oral and maxillofacial pain. These findings demonstrated the considerable role of SGC in orofacial pain, which was modulated by SP. Therefore, we suggest that the ERK1/2 and P38 MAPK pathways, as well as NK-1 receptors, may be potential therapeutic targets for the prevention and treatment of inflammatory orofacial pain.

## Materials and methods

### Animal preparation

All animal experimental procedures were approved by the Ethics Committee of West China Hospital of Stomatology Sichuan University (NO: WCCSIRB-D-2015-157). Sprague-Dawley (SD) rats (150–200 g) were housed in clean plastic cages on a 12-h light/dark cycle with unrestricted access to food and water. To establish the inflammation model in vivo, animals were anaesthetized with chloral hydrate (30 mL·kg^–1^, 10%, i.p.) and injected with complete Freund’s adjuvant (CFA, 0.05 mL, 1:1 oil/saline suspension) into the bilateral whisker pads, as described in our previous studies.^6^ The CFA-induced inflammation was verified with Evans blue dye (50 mg·mL^–1^, 1 mL·kg^–1^, i.v.). Physiological saline (0.05 mL, 0.9%) was injected into the bilateral whisker pads of SD rats to serve as the vehicle.

### Administration of drugs into the TG

To test the hypothesis that the MAPK pathway participates in inflammatory orofacial pain and the production of IL-1β and TNF-α from SGCs induced by SP, selective inhibitors were injected into the TG at a volume of 10 μL per side 30 min prior to CFA injection according to the method described in previous studies.^6,18,39,43,46^ Briefly, a midline skin incision (length, 1.5 cm) was performed from the head to the neck, after which a small hole (diameter, 1 mm) was drilled into the skull (2.6 mm anterior to lambda and 3.5 mm lateral to the midline) with a dental drill above the TG. A guide cannula (NeuroStar, German) was inserted through the hole to reach the TG, and the tip of the cannula was positioned 8.8-9.5 mm below the skull surface. The following drugs were used: L703, 606 (an antagonist of the NK-1 receptor, Sigma, USA), U0126 (an inhibitor of MAPK/ERK kinase [MEK] 1/2, Sigma, USA), SB203580 (an inhibitor of P38, Sigma, USA) and SP600125 (an inhibitor of JNK, Sigma, USA). These drugs were dissolved in dimethyl sulfoxide (DMSO; 0.5%, Sigma, USA) at a dose of 1 µg·µL^–1^. We used a solution including 0.5% DMSO as the vehicle. The dose and volume of the drugs mentioned above were based on previous studies.^39,43^

### Facial expression analysis

According to previous studies,^47,48^ facial expression analysis is commonly used to evaluate inflammatory allodynia in rats following the administration of different drugs. Rats (two at a time, *n* = 5 in each group) were placed in a box (20 cm × 15 cm × 10 cm) with transparent walls and no floor, and the box was framed on stainless steel shelves to ensure air circulation. Two digital video cameras were mounted (EOS D2000, Canon, Japan) on two sides of the box. The rats were videotaped for 30 min continuously in a quiet and softly lit environment. This procedure was implemented at baseline and repeated at each study time point (i.e., 1, 3, 5, and 7 d) after treatment. Then, the software (Rodent Face Finder® [RFF])^47^ was applied to capture 10 images of rat facial expressions from each videotaping session for scoring. The selected image files were randomly copied to Microsoft PowerPoint with one image per slide. The Rat Grimace Scale^47^ (RGS, including orbital tightening; nose/cheek flattening; ear changes; and whisker changes) scoring was used to evaluate the rat’s pain level. Orbital tightening involves the reduction of the eyelid area, the tight closing of the eyelids or the squeezing shut of the eyes (wrinkles around the eyes). Nose bulge refers to the circular skin extension visible on the bridge of the nose. Cheek bulge refers to the convex appearance of the cheek muscles (between the eyes and whiskers) protruding from their baseline position. Ear position means that the ear is pulled apart and moved backward from its baseline position or that a vertical ridge is formed owing to the tips of the ears being drawn back. Whisker changes occur when the whiskers move backwards from their baseline position, move against the face or move forward. Scores are assigned a value of 0 (not present), 1 (moderate) or 2 (severe) for each action unit in each photo. The mean RGS score of each rat at every observation time point was obtained to reflect the level of pain. Animals’ behaviour was analysed by researchers who were blinded to the experimental conditions.

### SGC culture and drug administration

To prove the effect of SP on SGCs in vitro, SGC cultures were established according to the method described by Capuano.^49^ We modified the protocol of TG isolation from SD rats (5–7 days) described in previous reports.^50^ Extracted TGs were washed using ice-cold Hanks’ balanced salt solution (pH = 7.4; Sigma, USA) and minced into small pieces under a dissecting microscope in modified α-MEM medium containing collagenase I (1 mg·mL^–1^, Solarbio, China) and trypsin (0.125%; Gibco, USA). At the end of the incubation, modified α-MEM medium containing foetal bovine serum (FBS, 10%; Gibco, Australia origin, USA) and antibiotics (100 μg·mL^–1^ penicillin and 100 μg·mL^–1^ streptomycin; Sigma, USA) was added to stop the digestion. After passage, purified SGCs were obtained and incubated at 37 °C and 5% CO_2_ in a humidified atmosphere. Immunofluorescence (IF) staining was carried out to prove that the cells were SGCs. More than 95% of the cells in the culture were SGCs based on images obtained using phase contrast microscopy as well as staining with the nuclear dye 4’,6-diamidino-2-phenylindole (DAPI; Abcam, Cambridge, UK) and antibodies directed against glutamine synthase (GS), a specific marker of SGCs. To further prove the important effects of SP on SGC activation and the production of IL-1β and TNF-α in vitro, we used SP (Sigma, USA, 1 μmol·L^–1^) alone and with co-application of L703, 606 (1 μmol·L^–1^) to stimulate SGCs for 24 h in order to generate the following groups: vehicle, SP and SP+L703, 606 (*n* = 6 in each group). To study the important effects of the MAPK pathway in the process of SP-induced SGC activation, the selective inhibitors U0126 (5 μmol·L^–1^), SB203580 (5 μmol·L^–1^) and SP600125 (5 μmol·L^–1^) were added to SGC cultures 30 min before SP administration. The SGCs were cultured for 24 h to obtain the following groups: SP+U0126, SP+SB203580 and SP+SP600125 (*n* = 6 in each group). Diluted DMSO (0.5%) in FBS-containing medium was used as a vehicle. The dose of drugs mentioned above was based on a previous study.^51^

### Immunofluorescence staining

For the animal models and SGC cultures, IF staining was applied to detect the expression of NK-1 receptors, phosphorylated (p)-ERK, p-P38 and p-JNK on SGCs. The dissected TGs were incubated in fixative (0.1 mol·L^–1^ phosphate buffer containing 4% paraformaldehyde, 4 h) and sucrose (30%, overnight). Frozen sections (10 μm thick) of TGs were cut on a cryostat (Leica, Germany) and mounted on saline-coated glass slides. Sections were processed as follows: rinsing with PBS (0.01 mol·L^–1^, 3 × , 5 min each), incubating with Triton X-100 (0.25%, 15 + min), blocking with goat serum (10%, 1 h, room temperature), and incubating with the primary antibodies (Table 1, overnight, 4 °C) and the secondary antibodies (Alexa Fluor® 594 and Alexa Fluor® 488, goat anti-rabbit or anti-mouse, 1:500, 1 h, 37 °C; Abcam, Cambridge, UK). Then, the cell nuclei were stained with DAPI (2–5 min). IF images were acquired by a microscope (Olympus, Japan). Similar procedures were also applied to SGC cultures after drug administration.

**Table 1:** Details of the primary antibodies used for IF and WB

Name	Product code	Host	Dilution	Company
GS	ab73593	Mouse/Rabbit	1:150(IF)	Abcam, Cambridge, UK
GFAP(50kD)	16825-1-AP	Rabbit	1:150(IF) 1:1 000(WB)	Proteintech, Wuhan, China
ERK1/2 (42, 44kD)	ab78953	Rabbit	1:1 000(WB)	Abcam, Cambridge, UK
p-ERK (PT202/PY204+PT185/PY187)(42, 44kD)	ab76299	Rabbit	1:200(IF) 1:5 000(WB)	Abcam, Cambridge, UK
P38(41kD)	ab32142	Rabbit	1:2 000(WB)	Abcam, Cambridge, UK
p-P38 (Thr180/Tyr182)(43kD)	4511	Rabbit	1:200(IF) 1:1 000(WB)	Cell Signaling Technology, Danvers, MA, USA
JNK(45-56kD)	51151-1-AP	Rabbit	1:500(WB)	Proteintech, Wuhan, China
p-JNK(T183+T221)(46-54kD)	ab124956	Rabbit	1:200(IF) 1:2 000(WB)	Abcam, Cambridge, UK
NK-1(35kD)	ab5060	Rabbit	1:200(IF)	Millipore, USA
IL-1β(17kD)	ab9722	Rabbit	1:1 000(WB)	Abcam, Cambridge, UK
TNF-α(25kD)	ab66579	Rabbit	1:1 000(WB)	Abcam, Cambridge, UK
β-actin	ab8226	Mouse	1:200(WB)	Abcam, Cambridge, UK

### Western blot assays

The isolated TGs were collected after homogenization and centrifugation to reveal the levels of glial fibrillary acidic protein (GFAP), ERK, p-ERK, P38, p-P38, JNK, and p-JNK by Western blot (WB). The protein concentration was measured with a DG-3022A microplate reader. Samples (40 µg) were mixed with Laemmli sample buffer (Amresco, USA), heated (95 °C, 10 min) and transferred onto PVDF membranes (Chemicon, Temecula, CA, USA) by a Bio-Rad semi-dry transfer apparatus. The membranes were soaked in blocking buffer (5% non-fat dry milk in PBS with 0.05% Tween-20, 1 h) and incubated with appropriate primary antibodies diluted in blocking buffer (Table 1, 4 °C, overnight). Afterwards, the membranes were washed in TBST and incubated in secondary antibodies diluted in blocking buffer (horseradish peroxidase [HRP]-conjugated, 1:50 000, anti-mouse/rat, 2 h, room temperature; Abcam, Cambridge, UK). After a series of washes, the membranes were stripped (15 min, room temperature) with Re-blot Plus Strong Solution (Chemicon, Temecula, CA, USA). We used β-actin as a loading control to normalize for protein loading. The membranes were visualized with a Fujifilm LAS-1000 Luminescent Image Analyzer (Fujifilm, Stamford, CT, USA), and the band optical density ratio was quantified by using BandScan software. SGC cultures were also subjected to WB guided by the protocol described above.

### Quantitative reverse transcription-polymerase chain reaction

Quantitative reverse transcription-polymerase chain reaction (RT-qPCR) was used to analyse the levels of IL-1β and TNF-α in SGCs treated with SP, antagonists of NK-1 receptors and inhibitors of MAPKs. The total RNA from the SGC cultures with different treatments was isolated using the Trizol method. In the reverse transcription reaction system, RNA was reverse transcribed into cDNA. cDNA was diluted 10 times and added to a real-time fluorescence quantitative PCR system (4 μL of cDNA, 0.4 μL of 10 μmol·L^–1^ forward primer, 0.4 μL of 10 μmol·L^–1^ reverse primer, 10 μL of SYBR Green Master Mix, 0.4 μL of 50 × ROX Reference Dye 2, and 4.8 μL of H_2_O; 40 cycles at 50 °C for 2 min, 95 °C for 10 min, 95 °C for 30 s, and 60 °C for 30 s) for the detection of mRNA expression. The primers for IL-1β, TNF-α and β-actin are listed in Table 2. The dissolution curves were plotted, and the final data were analysed with the 2^−ΔΔCt^ method.

**Table 2:** Details of the primer sequences used for RT-qPCR

Gene	Sequence(5′-3′)	Size
IL-1β	Forward: CCTGTGTGATGAAAGACGGC	218 bp
	Reverse: TATGTCCCGACCATTGCTGT	
TNF-α	Forward: CCGATTTGCCATTTCATACCAG	232 bp
	Reverse: TCACAGAGCAATGACTCCAAAG	
β-actin	Forward: CACGATGGAGGGGCCGGACTCATC	240 bp
	Reverse: TAAAGACCTCTATGCCAACACAGT	

### Enzyme-linked immunosorbent assays

SGC cultures were seeded in 6-well plates to investigate the expression of IL-1β and TNF-α after incubation under the following conditions: vehicle, SP, SP+L703, 606, SP+U0126, SP+SB203580 and SP+SP600125 (*n* = 6 in each group). The supernatants were collected, centrifuged (3 000 r·min^–1^, 10 min) and quantified using specific enzyme-linked immunosorbent assay (ELISA) kits (ab100768 and ab46070, Abcam, Cambridge, UK, examination range 68.59 pg·mL^–1^–50 000 pg·mL^–1^ and 31.25 pg·mL^–1^–1 000 pg·mL^–1^) and an ELISA reader (HTS700+, USA) according to the manufacturer’s instructions. The basal levels (257.23) were in the detectable (linear) range of the ELISA kits.

### Data analysis

All data are expressed as the mean ± SD and were analysed with Graph Pad Prism 6 and SPSS 20.0 by using one-way ANOVAs with Dunnett’s multiple comparison tests and one-sample *t*-tests, followed by Fisher’s least significant difference tests. The difference was considered statistically significant at *P* < 0.05.

## Supplementary information

EDITORIAL CERTIFICATE

Ethics approval and consent to participate

Author information

GS imaging

facial expression
