# Supplementary figures and images for: Activation of mitogen-activated protein kinases in satellite glial cells of the trigeminal ganglion contributes to substance P-mediated inflammatory pain
Source: Int J Oral Sci. 2019 Sep 10;11(3):24. doi: 10.1038/s41368-019-0055-0 (PMC6802677; doi:10.1038/s41368-019-0055-0)

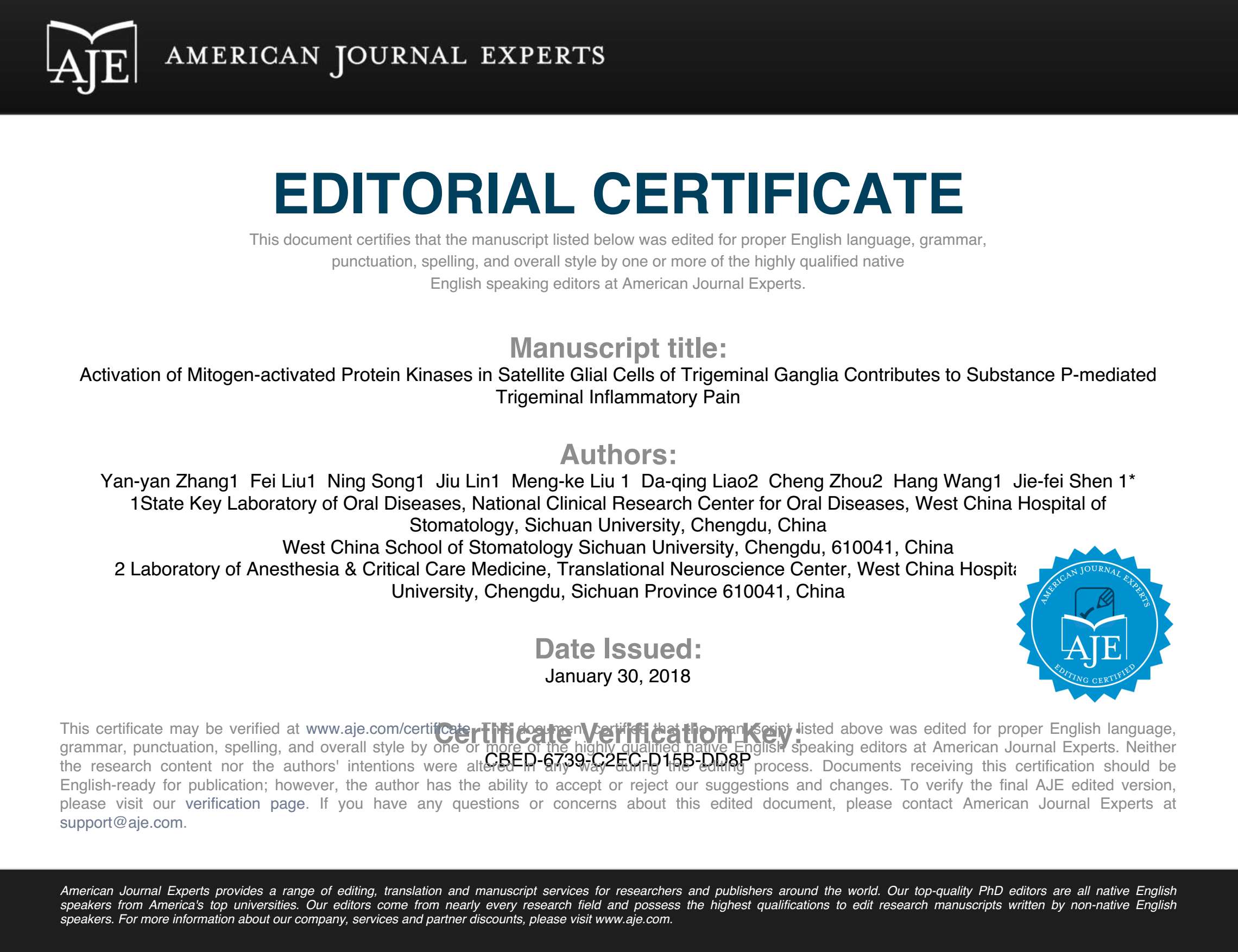

Supplement: Supplementary file 1 — EDITORIAL CERTIFICATE [file 41368_2019_55_MOESM1_ESM.docx]

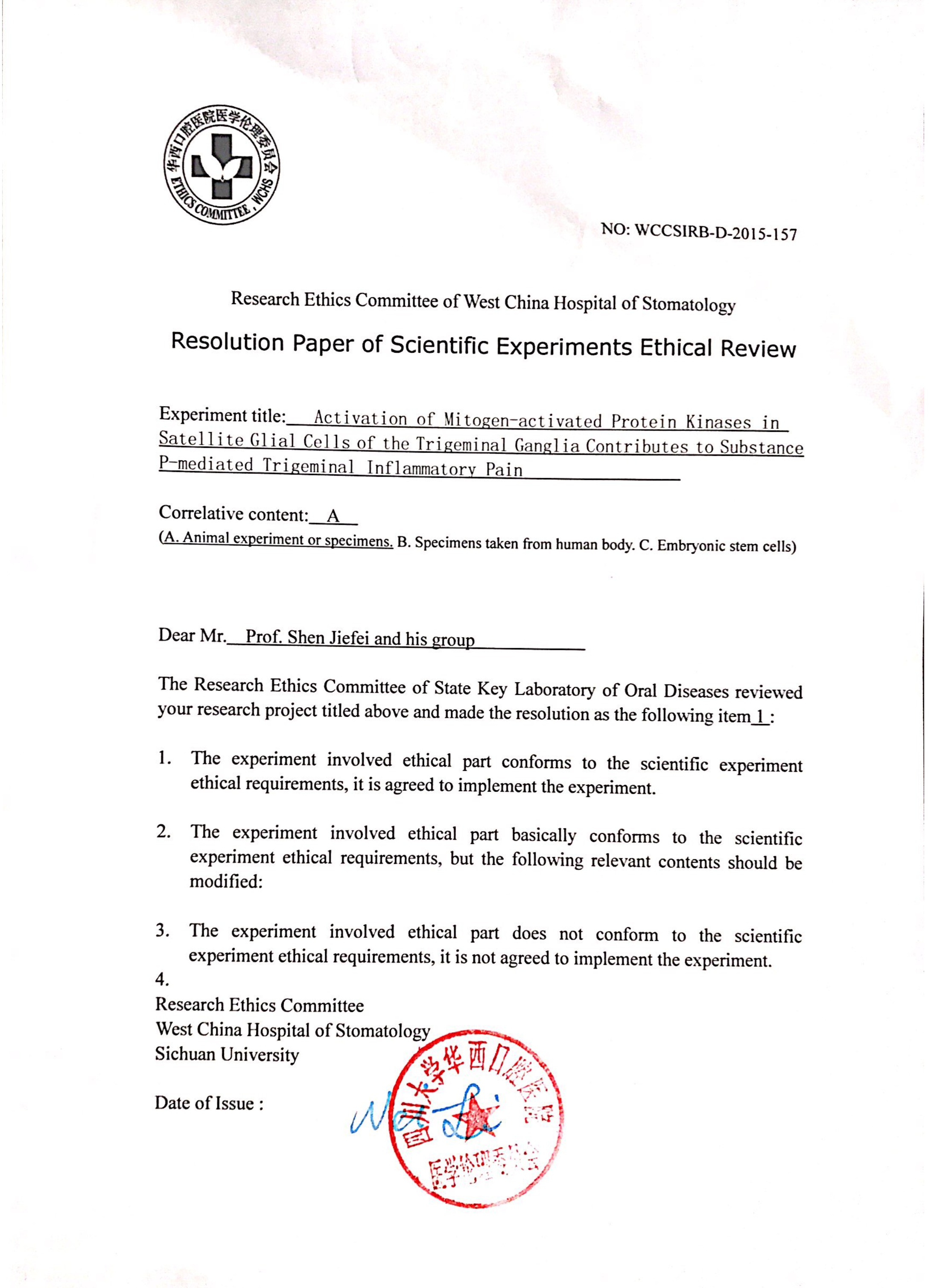

Supplement: Supplementary file 2 — Ethics approval and consent to participate [file 41368_2019_55_MOESM2_ESM.docx]

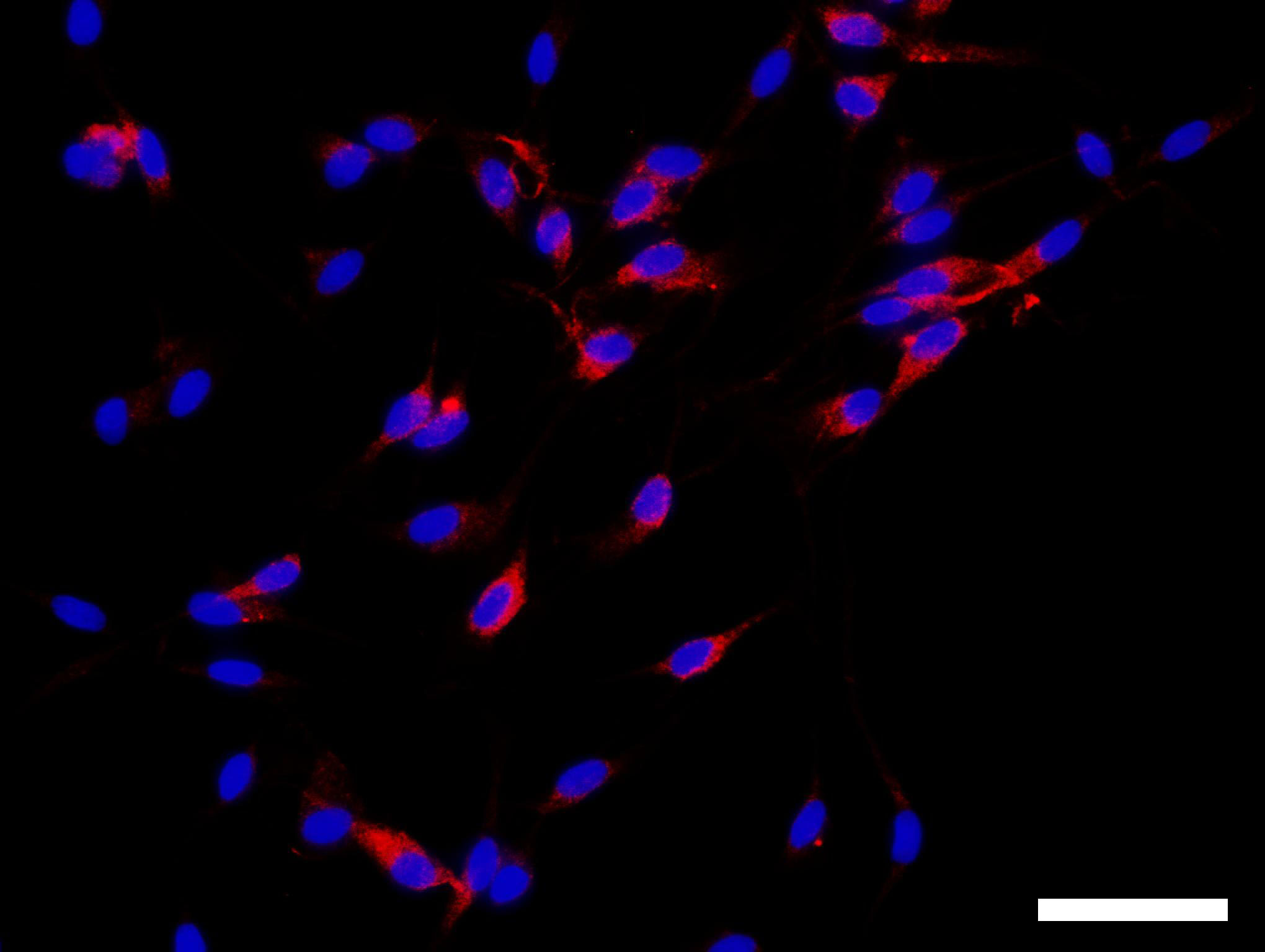

Supplement: Supplementary file 4 — GS imaging [file 41368_2019_55_MOESM4_ESM.jpg]
