# Supplementary material for: Activation of mitogen-activated protein kinases in satellite glial cells of the trigeminal ganglion contributes to substance P-mediated inflammatory pain
Source: Int J Oral Sci. 2019 Sep 10;11(3):24. doi: 10.1038/s41368-019-0055-0 (PMC6802677; doi:10.1038/s41368-019-0055-0)
Supplement: Supplementary file 3 — Author information [file 41368_2019_55_MOESM3_ESM.docx]

**Introduction to authors:**

Yan-yan Zhang^1^ Ning Song^1^ Fei Liu^1^ Jiu Lin^1^ Meng-ke Liu^1^ Chao-lan Huang^1^ Da-qing Liao^2^ Cheng Zhou^2^ Hang Wang^1^ Jie-fei Shen^1^*

1 State Key Laboratory of Oral Diseases, National Clinical Research Center for Oral Diseases, West China Hospital of Stomatology, Sichuan University, Chengdu, China

West China School of Stomatology Sichuan University, Chengdu, 610041, China

2 Laboratory of Anesthesia & Critical Care Medicine, Translational Neuroscience Center, West China Hospital of Sichuan University, Chengdu, Sichuan Province 610041, China

Yan-yan Zhang

*Email:* [*hxkqyanyanzhang@163.com*](mailto:hxkqyanyanzhang@163.com)

*West China Hospital of Stomatology*

*Sichuan University*

*No.14 Section 3, Renminnan Road*

*Chengdu, Sichuan Province, 610041*

*P.R. China*

Fei Liu

*Email:* [*aliyastomatology@163.com*](mailto:aliyastomatology@163.com)

*West China Hospital of Stomatology*

*Sichuan University*

*No.14 Section 3, Renminnan Road*

*Chengdu, Sichuan Province, 610041*

*P.R. China*

Ning Song

*Email:* *snxiaokeai@163.com*

*West China Hospital of Stomatology*

*Sichuan University*

*No.14 Section 3, Renminnan Road*

*Chengdu, Sichuan Province, 610041*

*P.R. China*

Jiu Lin

*Email:* [*799422754@qq.com*](mailto:799422754@qq.com)

*West China Hospital of Stomatology*

*Sichuan University*

*No.14 Section 3, Renminnan Road*

*Chengdu, Sichuan Province, 610041*

*P.R. China*

Meng-ke Liu

*Email:* [*mengke.liu@qq.com*](mailto:mengke.liu@qq.com)

*West China Hospital of Stomatology*

*Sichuan University*

*No.14 Section 3, Renminnan Road*

*Chengdu, Sichuan Province, 610041*

*P.R. China*

Chao-lan Huang

*Email:* *956565302@qq.com*

*West China Hospital of Stomatology*

*Sichuan University*

*No.14 Section 3, Renminnan Road*

*Chengdu, Sichuan Province, 610041*

*P.R. China*

Da-qing Liao

*Email: 281718556@qq.com*

Translational Neuroscience Center, West China Hospital of Sichuan University

*Tianfu Life Science Park*

*Chengdu, Sichuan Province, 610041*

*P.R. China*

Cheng Zhou

*Email:* [zhouc*@163.com*](mailto:zhouc@163.com)

Translational Neuroscience Center, West China Hospital of Sichuan University

*Tianfu Life Science Park*

*Chengdu, Sichuan Province, 610041*

*P.R. China*

Hang Wang

*Email:* *wanghang@scu.edu.cn*

*West China Hospital of Stomatology*

*Sichuan University*

*No.14 Section 3, Renminnan Road*

*Chengdu, Sichuan Province, 610041*

*P.R. China*

***Prof. Jie-Fei Shen, DDS & PhD***

*Email:* [*shenjiefei@scu.edu.cn*](mailto:shenjiefei@scu.edu.cn)

*West China Hospital of Stomatology*

*Sichuan University*

*No.14 Section 3, Renminnan Road*

*Chengdu, Sichuan Province, 610041*

*P.R. China*

*Telephone: +86-28-85501434*

*Email:* [*shenjiefei@scu.edu.cn*](mailto:shenjiefei@scu.edu.cn)

*Fax number: +86-28-85501434*
